# Towards FAIRification of sensitive and fragmented rare disease patient data: challenges and solutions in European reference network registries

**DOI:** 10.1186/s13023-022-02558-5

**Published:** 2022-12-14

**Authors:** Bruna dos Santos Vieira, César H. Bernabé, Shuxin Zhang, Haitham Abaza, Nirupama Benis, Alberto Cámara, Ronald Cornet, Clémence M. A. Le Cornec, Peter A. C. ’t Hoen, Franz Schaefer, K. Joeri van der Velde, Morris A. Swertz, Mark D. Wilkinson, Annika Jacobsen, Marco Roos

**Affiliations:** 1 Center for Molecular and Biomolecular Informatics, Radboud Institute for Molecular Life Sciences, Radboud University Medical Center, Nijmegen, The Netherlands; 2 Department of Medical Imaging, Radboud Institute for Health Sciences, Radboud University Medical Centre, Nijmegen, The Netherlands; 3 Department of Human Genetics, Leiden University Medical Center, Leiden, The Netherlands; 4 Department of Medical Informatics, Amsterdam UMC location University of Amsterdam, Meibergdreef 9, Amsterdam, The Netherlands; 5 Amsterdam Public Health, Methodology and Global Health, Amsterdam, The Netherlands; 6 Medical Informatics Group (MIG), University Hospital Frankfurt, Frankfurt, Germany; 7 Departamento de Biotecnología-Biología Vegetal, Escuela Técnica Superior de Ingeniería Agronómica, Alimentaria y de Biosistemas, Centro de Biotecnología y Genómica de Plantas (CBGP, UPM-INIA), Universidad Politécnica de Madrid (UPM) - Instituto Nacional de Investigación y Tecnología Agraria y Alimentaria (INIA), Madrid, Spain; 8 Division of Paediatric Nephrology, Centre for Paediatrics and Adolescent Medicine, University of Heidelberg, Heidelberg, Germany; 9 Genomics Coordination Center, University of Groningen and University Medical Center, Groningen, The Netherlands

**Keywords:** FAIR, Stewardship, Rare disease, Patient registry, Data steward

## Abstract

**Introduction:**

Rare disease patient data are typically sensitive, present in multiple registries controlled by different custodians, and non-interoperable. Making these data Findable, Accessible, Interoperable, and Reusable (FAIR) for humans and machines at source enables federated discovery and analysis across data custodians. This facilitates accurate diagnosis, optimal clinical management, and personalised treatments. In Europe, twenty-four European Reference Networks (ERNs) work on rare disease registries in different clinical domains. The process and the implementation choices for making data FAIR (‘FAIRification’) differ among ERN registries. For example, registries use different software systems and are subject to different legal regulations. To support the ERNs in making informed decisions and to harmonise FAIRification, the FAIRification steward team was established to work as liaisons between ERNs and researchers from the European Joint Programme on Rare Diseases.

**Results:**

The FAIRification steward team inventoried the FAIRification challenges of the ERN registries and proposed solutions collectively with involved stakeholders to address them. Ninety-eight FAIRification challenges from 24 ERNs’ registries were collected and categorised into “training” (31), “community” (9), “modelling” (12), “implementation” (26), and “legal” (20). After curating and aggregating highly similar challenges, 41 unique FAIRification challenges remained. The two categories with the most challenges were “training” (15) and “implementation” (9), followed by “community” (7), and then “modelling” (5) and “legal” (5). To address all challenges, eleven types of solutions were proposed. Among them, the provision of guidelines and the organisation of training activities resolved the “training” challenges, which ranged from less-technical “coffee-rounds” to technical workshops, from informal FAIR Games to formal hackathons. Obtaining implementation support from technical experts was the solution type for tackling the “implementation” challenges.

**Conclusion:**

This work shows that a dedicated team of FAIR data stewards is an asset for harmonising the various processes of making data FAIR in a large organisation with multiple stakeholders. Additionally, multi-levelled training activities are required to accommodate the diverse needs of the ERNs. Finally, the lessons learned from the experience of the FAIRification steward team described in this paper may help to increase FAIR awareness and provide insights into FAIRification challenges and solutions of rare disease registries.

**Supplementary Information:**

The online version contains supplementary material available at 10.1186/s13023-022-02558-5.

## Introduction

Rare diseases (RDs) are defined as life-threatening or chronically debilitating conditions that affect a low percentage of the population. In Europe, diseases are considered “rare” when their prevalence is less than 5 per 10,000 people [1]. Their low prevalence means that RD patient data is scarce and fragmented. Consequently, it is difficult to access sufficient data to support, for instance, research, drug development and improvements in outpatient care. The Orphanet, the National Organisation for Rare Diseases (NORD) [2], and other initiatives around the world have deemed it important to improve collaboration for research [3] and Open Science for RD [4]. Such initiatives make it easier for people with RDs to share their data. In fact, the importance of data sharing is consistently emphasised by RD patients themselves [5]. To help with research on RDs, the European Joint Programme on Rare Diseases (EJP RD) was set up in 2018 [6]. The programme aims to solve the problem of fragmented information and to build a research ecosystem that makes the best use of data and resources, thus benefiting people with RDs. The EJP RD project collaborates directly with the 24 European Reference Networks (ERNs) [7], which involve more than 900 highly specialised healthcare units from more than 130 institutions in 35 countries [6]. Each ERN works on a subset of RDs and maintains registries of varying complexity. Some ERNs have a single centralised registry to which participating healthcare providers submit data, whereas others have registries established in their participating institutes, where each institute collects and maintains its data.

Unfortunately, because each ERN collects unique data, there are wide variations in terms of content, format, and language across their RD registries. This heterogeneity makes it virtually impossible to jointly analyse ERN data, wasting considerable time and effort for data analysts and affecting any large-scale research project aimed at improving RD patient care. For instance, counts of patients with similar symptoms, treatments for similar symptoms across different geographic regions, or time-to-diagnosis cannot be produced by a simple query across all registries. A patient representative searching for “genomes pertaining to a rare disease profile not yet classified as such” or a researcher analysing “observed phenotypes of citizens with the same genetic profile” with the aim to “identify correlations with regional factors” are examples of more complex queries that can be executed on multiple resources across institutes and countries, the premises of which, however, is to make data Findable, Accessible, Interoperable, and Reusable (FAIR). It is, therefore, crucial to improve the Findability, Accessibility, Interoperability and Reusability (FAIRness or FAIR ‘maturity’) of the data collected in the RD registries of the 24 ERNs, for both humans and machines, as stated in the FAIR Guiding Principles [8]. When data are FAIR, they can be queried in an unambiguous and federated way, globally (if appropriate reuse conditions are met) without leaving its premises [9, 10]. In addition, an ecosystem based on FAIR principles adapts its functionality to its sources, because each source is self-explanatory.

Various methods can be applied for making data FAIR (also referred to as ‘FAIRification’) among the 24 ERNs, which contributes to diverging FAIRification methods and implementation choices throughout the network of ERNs. These differences are due to 1) different requirements and objectives (e.g., an initial focus on legal aspects, or a focus on internal queriability), 2) different software systems and tools (e.g., an Electronic Data Capture (EDC) system, the lack of license for a specific ontology), 3) different disease domains (e.g., rare types of cancer, bone diseases), and 4) different jurisdictions (e.g., different laws between centres/countries). Applying different FAIRification methods theoretically still leads to interoperable solutions by definition, but overall, the process is not efficient for a community. Thus, harmonisation of methods and definitions and sharing of best practices would be beneficial to maximise the efficiency and benefit of FAIRification for all stakeholders.

Data can be made FAIR retrospectively, often long after they were collected, which may require extensive efforts to understand the meaning of the data [11–13]. Data can also be made FAIR when they are being collected, where the FAIRification steps are embedded in the data collection tool [14]. The latter was implemented for a VASCERN ERN registry, where data are made FAIR automatically and in real-time upon collection [15]. This FAIRification workflow can be reused by other ERNs across data collection platforms. Nevertheless, there is a need to guide the ERNs in achieving higher efficiency by aligning their implementation choices regarding tools (e.g., EDC software), standards (e.g., data representation syntaxes, ontologies), and legal decisions (e.g., sending data to a central registry in a different country versus several hospitals with their own FAIR databases, informed consent forms, data access policies, data processing and sharing agreements).

To harmonise FAIRification across ERN RD patient registries, a FAIRification steward team was established to act as liaisons between the ERNs and FAIR experts. These liaisons, supported by the EJP RD, provide a unique opportunity to investigate the ERNs’ understanding and application of the FAIR principles to enable the use of data across international borders in the RD field. This work aims to 1) identify the challenges in FAIRifying RD registries and 2) support European-wide harmonised FAIRification by proposing solutions in the RD field.

## Methods

### Organisation of the FAIRification steward team

**Fig. 1:**
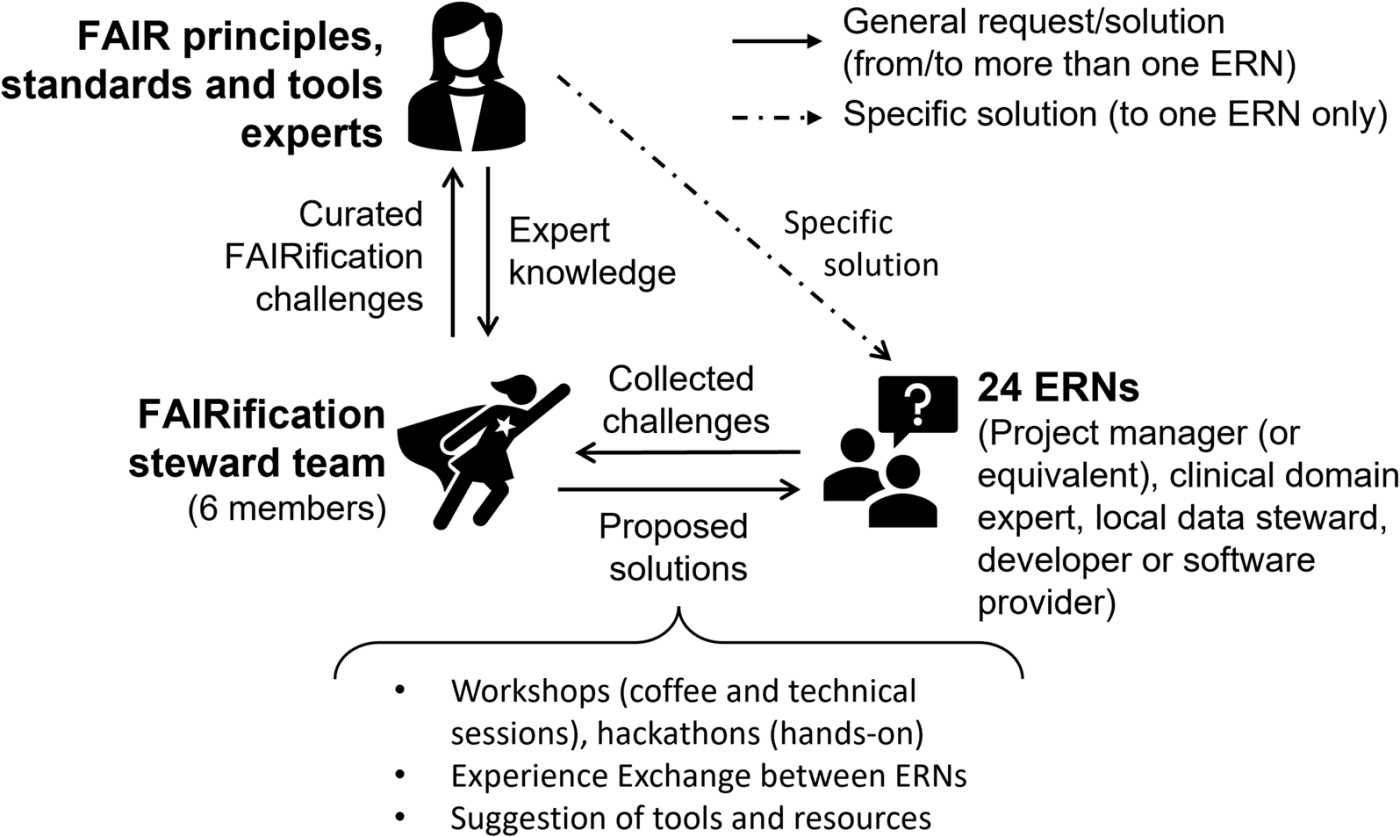
FAIRification steward team, EJP RD FAIR (principles, standards, and tools) experts, and European Reference Networks (ERNs) in a three-party interaction map. The FAIRification steward team works as liaisons between ERNs and EJP RD FAIR experts, collecting FAIRification challenges from ERNs, curating these challenges, providing them to experts, and returning consolidated knowledge from the experts to ERNs as proposed solutions. For single ERN requests, the team creates Expert-ERN communication channels (dashed line). The ERN team includes a project manager (or equivalent), a local data steward, and a developer (or software provider). The set of proposed solutions comprises workshops, where standards or tools are presented by experts; hackathons, where developers can try different tools themselves in a hands-on fashion; experience exchange between ERNs; and suggestions of existing implementations, tools, and resources

The EJP RD FAIRification steward team was established on July 10th, 2020, to support and ensure harmonised FAIRification of ERN RD patient registries. The team is composed of six FAIR data stewards with different scientific backgrounds (biomedical science, software development, hospital management, public health, engineering) and education levels (BSc, MSc, and PhD). As illustrated in Fig. 1, the FAIR data stewards facilitate the communication between ERNs and EJP RD FAIR experts. Each FAIR data steward collects FAIRification challenges from the ERNs they are assigned to. Then, the team curates these challenges and submits them to the FAIR experts, who provide the knowledge that is needed for proposing solutions. The team conveys the challenges requiring customised and ongoing support for a single ERN to the relevant experts and requests specific solutions.

Each ERN formed a core FAIRification team, including a project manager or equivalent (e.g., data manager, registry manager), a clinical domain expert, a local data steward, and a developer. The last could be replaced by the hired EDC company's programming support. Each FAIR data steward supports four ERNs and is the backup for four other ERNs. The communication channels between each ERN and their FAIR data steward were established in a first introduction meeting, and thereafter maintained in follow up meetings on demand.

### Identification of the FAIRification challenges

We identified the FAIRification challenges of the ERN RD patient registries in two main steps: collection of challenges and curation of challenges. The second step consists of three sub-steps: categorisation, rephrasing, and merging of challenges. These are further detailed in this subsection.

Firstly, the FAIR data stewards collected the challenges that ERNs had with making their RD patient registries FAIR based on an initial set of 77 tools and standards identified by EJP RD FAIR experts. The implementation status of each standard or tool was identified for each ERN (“Implemented”, “Plans to Implement”, “Need Expert Help”, “Implementing Assisted by Expert” or “Non-Applicable”), as exemplified in Table 1. Note that additional tools and standards could be added where applicable, as disclaimed in the document. Questions and implementation details specific to a tool or standard were recorded for each ERN and used as the main input for the FAIRification challenges. These data were collected by the FAIR data stewards while meeting with ERNs and stored in a persistent and traceable document. To preserve privacy, access to this data is restricted to the associated EJP RD FAIR experts and FAIR data stewards. The FAIR data stewards continued to communicate with ERNs regularly to provide feedback and follow-up on their questions, which could lead to additional FAIRification challenges.

**Table 1:** An excerpt of the document used to collect the implementation status of each tool and standard for each ERN

Function	Tool/standard name	ERN registry implementation status
Data model	CDE semantic model	*Implemented*
Set of data elements	Common data elements JRC	*Implemented*
Genes Ontology	HGNC	*Plans to Implement*
Genes Ontology	HUGO	*Non-Applicable*
Variant Ontology	HGVS	*Plans to Implement*
Phenotype Ontology	HPO	*Needs expert help (see methods)*
International Classification of Diseases	ICD-10	*Non-Applicable*
International Classification of Diseases	ICD-11	*Implemented*
Minimum Information About Biobank Data Sharing	MIABIS	*Implementing assisted by expert*

The first column describes functions related to tools and standards which are listed in the second column. The last column tracks the implementation status of each tool or standard (“Implemented”, “Plans to Implement”, “Need Expert Help”, “Implementing Assisted by Expert” or “Non-Applicable”). The references to the tools can be found in the template of the Additional file 1The first column describes functions related to tools and standards which are listed in the second column. The last column tracks the implementation status of each tool or standard (“Implemented”, “Plans to Implement”, “Need Expert Help”, “Implementing Assisted by Expert” or “Non-Applicable”). The references to the tools can be found in the template of the Additional file 1

Secondly, all FAIRification challenges collected in the previous step by December 31st, 2020, were categorised, rephrased, and merged. All FAIRification challenges were categorised by: (1) “training”, specifying the need for training on a specific technology or concept; (2) “community”, requiring peer experience exchange; (3) “modelling”, relating to (meta)data models or conceptual modelling activities; (4) “implementation”, requiring programming expertise, such as the implementation of data exchange interfaces between systems; and (5) “legal”, describing questions about data sharing and reuse agreements, informed consent, or any related services (e.g., patient informed consent form). These categories were defined by the FAIRification steward team based on the commonalities identified among the challenges. The categories and their definitions are summarised in Table 2. With this categorisation, we standardised the presentation of common solutions to avoid the need for repeated referrals to experts.

**Table 2:** List of categories and their definitions

Category	Definition
Training	Challenges related to inquiries for more information on a specific tool, standard, or a general concept
Community	Challenges involving activities of peers in the same community to achieve reuse and prevent duplicated effort
Modelling	Challenges involving the conceptualisation of data into data elements and bindings of standardised vocabularies to these data elements
Implementation	Challenges involving implementation of a specific tool or standard
Legal	Challenges related to inquiries about data sharing and reuse agreements, informed consent, or implementation of related services

Five categories were created to organise the FAIRification challenges of RD patient registries. The categories reflect the nature of the challenges: the need for training, to learn from others, information about modelling, implementation, or legal aspects

The FAIRification challenges after categorisation were rephrased and merged based on their content and commonalities. For instance, the two example challenges “We need hands-on help to implement the Common Data Element (CDE) [16] in REDCap (Research Electronic Data Capture) [17]” and “How can the CDE Semantic Model be implemented in Marvin XClinical [18]?” could be merged to one curated challenge “How to implement the CDE model [19] in my EDC system?”.

All processes, i.e., categorisation, rephrasing, and merging, were at least reviewed by two independent reviewers. The FAIRification challenges that result from this processing are referred to as curated FAIRification challenges. The remaining inconsistencies were resolved in discussions with the entire team and, upon need, with EJP RD FAIR experts.

### Proposing solutions to the FAIRification challenges

The FAIR data stewards defined solutions to the curated FAIRification challenges in collaboration with different stakeholders. The five stakeholder groups who contributed to the development of these solutions were: (1) ERN representatives, (2) EJP RD FAIR (principles, standards, and/or tools) experts, (3) EJP RD coordinators, (4) Joint Research Centre, and (5) software developers and providers. To maximise efficiency, we defined solutions capable of addressing the highest number of challenges simultaneously. For the challenges that could be solved using readily available single solutions, we directly contacted the relevant stakeholders. Further, for the challenges that required novel solutions to be developed, the recombination of existing solutions, a long-term effort, or the participation of multiple parties, we arranged various types of activities that allowed for brainstorming for all stakeholders including ERNs.

## Results

Here we present the work by the EJP RD FAIRification steward team to support the FAIRification of ERN RD patient registries. This includes the list of identified FAIRification challenges and proposed solutions to the ERNs. The solutions were reused or developed with input from multiple internal and external stakeholders to ensure convergence.

### Overview of FAIRification Challenges

Ninety-eight FAIRification challenges were collected from all 24 ERNs. Their respective counts for each category before “original”) and after curation are shown in Table 3. The most common category was “training” (31) while the least common was “community” (9). The “implementation” category contained 26 challenges, “legal” contained 20, and finally“modelling” contained 12. More details on all original and curated challenges can be found in the [see Additional file 2].

**Table 3:** The number of FAIRification challenges for each category (training, community, modelling, implementation and legal) defined in our approach

FAIRification challenges	Categories				
	Train.	Comm.	Model.	Impl.	Legal
Original (98)	31	9	12	26	20
Curated (41)	15	7	5	9	5

The second and third rows show the number of challenges before and after curation, respectively

After curation, the total number of challenges was reduced to 41. The “implementation” category had the biggest reduction (from 26 to 9). The “training” category was reduced from 31 to 15, “legal” from 20 to 5, “modelling” from 12 to 5, and “community” from 9 to 7. The “training” and “implementation” categories remained the most and second most common categories, respectively. On the other hand, “modelling” and “legal” were the categories with the lowest number of challenges after curation.

The fifteen curated “training” challenges were either related to a tool or standard, for example, CDEs, CDE semantic model [19], mapping languages, FAIR Data Point, registration of registries through the European Rare Disease Registry Infrastructure (ERDRI) [20], informed consent, pseudonymisation, and query (see Table 4). “More information on semantic data model”, and “More information on the FAIR Data Point (FDP)” are examples of “training” challenges.

**Table 4:** A summary of the identified training FAIRification challenges and proposed solutions

Curated training FAIRification challenges	Specific solution
More information on ERDRI (added value, utility)	“Coffee rounds”” (ERDRI, Orphacodes, EUPID, Practical requirements, Practical implementation, Resource finder, Informed Consent, Disability and QoL)”
Documentation and specification of CDEs	Documentation of Semantic CDE Model
More information on CDE model (e.g. what is does, what is the added value, what would be the effort to implement it)	
More information on ADA-M and machine readable consent	ERN Technical Workshops (Semantic CDE Model, EJP RD Metadata Model, EUPID API, Data formats and mapping languages, Phenopackets, Query builder, Orphacodes, DCDEs, PROMs)
More information on Beacon 2.0 (added value, utility, how to implement it)	
More information on EJP-RD Metadata Model (what it does, what is the added value)	
More information on EUPID (e.g. licenses, costs)	
More information on FAIR Data Point (how will it work)	
More information on Phenopackets (utility)	
More information on Querying	
More information on RDF Mapping Languages	
What interoperability impact difference would be between using CDE Model and OMOP-CMD?	
More information on FAIR	Rome Summer School
Ground rules for interoperability (e.g., terminology, personnel, connectivity mechanism, API definition sets, diagrams, and technology specification)	Virtual Platform Specification (VIPS)
More information on EJP RD Virtual Platform	

The first column lists the curated challenge, while the second describes the specific solution used to address that

The nine curated “implementation” challenges (see Table 5) were not only related to the tools and standards mentioned above but also “data format”, to which 11 original challenges were merged. One example of these original challenges was “What are the recommendations for data formats for the EJP RD Virtual Platform?”.

**Table 5:** A summary of the identified implementation FAIRification challenges and proposed solutions

Curated Implementation FAIRification challenges	Specific solution
How can I use the iCRF generator tool?	Experts from the CDE Modelling group for data conversion
How to implement the CDE model in different EDC systems?	
Advice on data representation languages	
How to create RDF triples from a SQL database?	
How to integrate FDP in a registry?	
Is there a common template for excel import/exports (of the CDEs?)?	
Is there a template for batch import of metadata elements into ERDRI.MDR?	Experts from the EU RD Platform for findability of registries
How can the EJP RD metadata model be implemented?	Experts from the Metadata Modelling group for metadata conversion
How can the query builder tool be implemented on my system?	Experts from the Query Builder group for data querying

The first column lists the curated challenge, while the second describes the specific solution used to address that

The seven curated “community” challenges were related to the need for individual ERNs to learn from other ERNs (see Table 6). For instance, data sharing policies differ between healthcare providers at both the national and international levels, prompting ERNs to inquire about how the other ERNs dealt with such constraints.

**Table 6:** A summary of the identified community FAIRification challenges and proposed solutions

Curated community FAIRification challenges	Specific solution
How other ERNs annotate disability questionnaire?	Disability survey
Exchange of experiences between ERNs registries	Exchange of FAIR experience
What tools and standards do other ERNs use?	
Learn from advanced registries with examples	Exchange of information on a regular basis
How do other ERNs share data?	
How do other ERNs collect the following CDEs: 2.1 Date of Birth, 6.1 Diagnosis and 6.2 Genetic Diagnosis?	Share data dictionaries for identifying DCDEs
What database templates do other ERNs use?	

The first column lists the curated challenge, while the second describes the specific solution used to address that

The five curated “modelling” challenges (see Table 7) were all related to the CDEs but from different perspectives: how to interpret non-applicable CDEs (3); how to model non-compliant CDEs (3); how to interpret poorly defined CDEs (1); which ontology is recommended for a certain case (4); what if Orphanet is not sufficient for some RDs (1). For example, the data element “date of birth” is not allowed to be recorded due to national regulations, so only “birth year” is recorded. Another example is the WHO (World Health Organisation) Disability Assessment Schedule (WHODAS) [21]. It is a recommended standard for the data element “disability score”, but it does not apply to paediatric patients.

**Table 7:** A summary of the identified modelling FAIRification challenges and proposed solutions

Curated modelling FAIRification challenges	Specific solution
Which ontology is recommended for [X]?	CDE Modelling group
Are non-applicable CDEs mandatory?	Experts from the JRC for Common Data Elements
What if collected data do not follow the formats required in CDEs?	
What if the CDE [X] is not well-defined?	
What if Orphacode is not sufficient for [X] diseases?	Experts from the Orphanet group for Orphacode

The first column lists the curated challenge, while the second describes the specific solution used to address that

The five curated “legal” challenges (see Table 8) were related to legal concerns of the pseudonymisation tool (4) and its implementation (11), informed consent (1) and its machine-readable implementation (1), and data processing agreements and access policies (3).

**Table 8:** A summary of the identified legal FAIRification challenges and proposed solutions

Curated legal FAIRification challenges	Specific solution
How can machine-readable information consent be modelled?	EJP RD Consent Template
Which consent form should be used?	
European level guidance on: Data Processing Agreements per database and countries; Agreements between EDC software and Hospitals that include multiple ERNs; ERNs Consortium agreement; Legal issues between countries.	ERICA project
How to implement EUPID within a registry?	Experts from the pseudonymisation tool group
What are the legal concerns about the EUPID implementation?	

The first column lists the curated challenge, while the second describes the specific solution used to address that

### Overview of proposed solutions

Eleven types of solutions were proposed to address the different categories of FAIRification challenges (see Table 4). To address the “training” challenges, two types of solutions were proposed: (1) provide guidelines, and (2) organise training events. For the guidelines, EJP RD has created a list of deliverables [22] to establish concrete specifications that ERNs can adhere to. These deliverables include, for example, a report on the core set of unified FAIR data standards. For the training events, seven “coffee rounds” and eleven “technical workshops” [23] were organised. “Coffee rounds” were aimed to provide basic knowledge of tools or standards to a non-technical audience, whereas the “technical workshops” were designed to provide a more in-depth and technical understanding of how to implement a tool or standard. Through online surveys, the ERNs determined the topics and prioritised the order of the “coffee rounds” and “technical workshops”. The coffee round “Introduction of the Orphanet nomenclature and the ORPHAcodes”, for example, introduced the concept of ORPHAcodes [24], clarified its objectives, and explained the benefits of its use. The “ORPHAcodes” technical workshop was organised to demonstrate how the standard could be implemented within an RD registry. Many of the “training” FAIRification challenges were addressed in the International Summer School on Rare Disease Registries and FAIRification of Data [25]. In this event, both FAIR data stewards and FAIR experts (EJP RD and external) were trainers.

Three solutions were proposed to address the “community” challenges: (1) survey ERNs and report on a specific challenge, (2) arrange experience exchange meetings, and (3) share information (see Table 6). In the first solution, a FAIR data steward got a request from their assigned ERNs on how peer ERNs resolved a particular challenge. For instance, WHODAS does not consider paediatric patients, which is insufficient to capture disability information in the domain of some ERNs. They then inquired whether other ERNs used alternative tools to assess the disability of paediatric patients in their registry. The FAIRification steward team then developed a survey on this request, which was disseminated to all ERNs by their assigned steward, respectively. The survey results were recorded and made available to ERNs upon request. The solutions were recorded to be used as input for the development of guidance tools.

In the second solution, the FAIR data stewards arranged experience exchange meetings between two ERNs when one ERN wanted to learn from (or collaborate with) another ERN at a more advanced stage in the FAIRification of their registry. Knowledge exchange between ERNs also contributes to the harmonisation of the FAIRification solutions across them. As an example, an exchange meeting was held between two closely collaborating ERNs that use the same platform and methods with common research interests in related diseases. This enables them to communicate with the FAIRification steward team as a single entity. Another example is an exchange meeting held between two advanced ERNs who wanted to exchange FAIRification experience and sought further collaboration regarding Patient-Reported Outcomes (PROs).

For the third solution, information sharing among ERNs was harmonised by FAIR data stewards. A typical example of this was that ERNs shared their data dictionaries (e.g., e-REC form in EuRRECa [26]) with the FAIRification steward team. Each ERN-specific data dictionary lists data elements to be collected in their registries together with definitions and accepted values.

The solution proposed to all “implementation” challenges is “to get implementation support from relevant experts”, regardless of the tools or standards in question. The FAIR data stewards organised hackathons to define reference software implementations across ERNs (e.g., Implementation CDE Semantic Model for ERNs EDC providers [27]). These hackathons were held for individual ERNs, where FAIR experts gave hands-on support to a specific FAIRification challenge of an individual ERN.

Two types of solutions were proposed to address “modelling” challenges: 1) get modelling advice from relevant experts, and 2) organise a modelling group (see Table 7). The first solution mainly resolved challenges about ORPHAcodes [28] and CDEs, e.g., “how to model diseases that are not captured by ORPHAcodes”, and “how do we interpret CDEs that are not well-defined”. The second solution aimed to establish a dedicated modelling group for modelling discussions. The EJP RD CDE modelling group focuses on semantic data modelling (initially for CDEs, but now for other modelling needs) and provides support for addressing “modelling” challenges.

Three solutions were proposed to tackle challenges with informed consent, pseudonymisation, and data sharing policies in the “legal” category: (1) develop a generic consent form, (2) get implementation support from experts who develop the pseudonymisation tool, and (3) reach data processing agreements and data sharing policies (see Table 8). In the EJP RD, a generic consent form [29] involved European institutions. This generic consent form was subsequently translated into 25 national languages. The European Rare Disease Research Coordination and Support Action (ERICA) [30] Work Package 2 was created to support the ERNs in all aspects related to data collection, integration and sharing, including ethical requirements.

## Discussions

The proposed solutions to the FAIRification challenges presented in this paper contribute to increased harmonisation of FAIRification implementation decisions across ERN RD patient registries. Through workshops, the ERNs were not only connected to experts but also to other ERNs. These workshops created a collaborative environment for the exchange of ideas and the implementation of solutions. The following subsection presents discussions on diversity in the FAIRification challenges, the strengths and weaknesses, lessons learned from the FAIRification steward team, and future work.

### Diversity in FAIRification challenges

The notable reduction in the total number of FAIRification challenges following curation from 98 to 41 (see Table 2) indicates that there are a considerable number of common ERN concerns (57), but also highlights the diversity among the challenges (41). The largest number (15) of curated “training” challenges reveals a gap in knowledge and a lack of access to training in the distinct aspects of FAIRification. Advice on data representation languages, the CDE semantic model, the EJP RD metadata model [31], mapping languages [32], and the pseudonymisation tool [33] are all examples of frequently encountered “training” challenges by ERNs. The other four categories are less diverse with their number of challenges ranging from 5 to 9, which becomes more evident in the “implementation” category, reducing from 26 to 9 curated challenges.

“Legal” challenges are mainly attributed to (1) the variation of legal documents required to collect, process, and grant access to the data [34], and (2) the lack of awareness of EU-wide pseudonymisation tools. The variation of legal documents exists because of the country-specific legislation and different interpretations and applications of GDPR (General Data Protection Regulation) [35, 36]. Some countries even request additional safeguards for sensitive data, which increases the complexity of establishing a patient registry. The lack of awareness of using EU-wide pseudonymisation tools was another practical issue that resulted in some of the “legal” challenges. Given that some ERNs already had an internal pseudonymisation system in place, they questioned the added value of using an additional pseudonymisation tool and were concerned about the cost of re-assigning pseudonyms to existing patient records. Currently, the European Joint Research Centre (JRC) is working on the development of an EU-wide pseudonymisation tool to be reused by the ERNs.

The “community” challenges refer to how others use standards and tools. The fact that ERNs look for reusing peer solutions fosters convergence and interoperability. This is a positive observation because standards are only interoperable when used across organisations [37]. In fact, the third foundational principle of FAIR, *Interoperability*, is the most challenging one to be realised [38], and consequently, requires considerable effort [39]. Once the community standards are agreed upon, the reusability of data is facilitated, contributing to a sustainable scientific environment [40]. Convergence over the tools and standards used to promote interoperability within the community is necessary and will benefit new registries in general. Thus, it enables the RD community to define its FAIR Implementation Profile [40], a list of community-supported choices that promote convergence for FAIRification. In general, interoperability issues extended beyond technical FAIR standards to include legal and modelling considerations. For instance, country-specific legislation may prohibit the collection of certain data elements, thereby directly impacting modelling and thus the data sharing capabilities between registries from different countries. To support these legal and ethical challenges, EJP RD offers a helpdesk and an AREB (Advisory Regulatory Ethics Board) [41] office.

### Strengths and weaknesses of the approach

By forming a team of FAIR data stewards from diverse backgrounds we were able to harmonise the disparate FAIRification procedures of RD registries. The workload was efficiently balanced among the stewards, enabling effective communication with ERNs. This consulting experience resulted in increased networking, convergence, and dissemination of knowledge. Besides, the FAIRification challenges of the ERNs were gleaned as first-hand information by the FAIR data stewards. Therefore, the challenges could accurately reflect the actual issues faced by RD registries in their EU-wide FAIRification and serve as valuable information for decision-making at the project level.

When compared to our previous FAIRification experience involving a single FAIR data steward [14], a team supported FAIRification effort resulted in a more robust approach. First, the diverse backgrounds and the collaboration among team members facilitated experience exchange and FAIRification discussions. This has enabled the stewards to scrutinise FAIRification challenges from a variety of angles, resulting in the development of a collection of diverse solutions. Secondly, such team-based support enables the stewards to maintain a consistent pace in the communications with the ERNs, for example by the support of backup stewards, as one person could assist an overwhelmed teammate when necessary.

Since each of the FAIR data stewards may have had slightly different discussions in their regular meetings with the ERNs, there may have been differences in the way each ERN described their challenges. However, this bias was reduced by using the initial set of tools and standards (see the Methods Section) as a starting point for these discussions. The same is true for the interpretation of the original FAIRification challenges by each of the FAIR data stewards. The rephrasing style may have varied and influenced the final number of curated challenges. To mitigate this problem, we performed cross-checking between pairs of stewards, so one could validate the rephrasing and merging of the other.

Nonetheless, the significance and implications of our findings, particularly in the progress of RD registry FAIRification, reinforce the importance of this type of work. The steps taken by the FAIRification steward team to communicate, collect information, and identify solutions can, therefore, be reused as guidance for other FAIR project management in general. In addition, the sustainability of any approach developed in the EJP RD is a core value of the project that also concerns the FAIRification steward team. In September 2021 during the EJP RD general assembly, a workshop [42] on the sustainability of the FAIRification steward service was held, and it was concluded that this EU-wide service should be continued and made available to other types of resources apart from registries.

### Lessons learned

The unique experiences from the interaction between the FAIR data stewards and the diverse RD registries are summarised below:

- *Clarify FAIRification goals before implementation.* Available FAIRification workflows recommend that defining the FAIRification goal(s) is the first key step in FAIRification [11, 14]. Nonetheless, some of the RD registries have not completed this step yet. When defining clear FAIRification objectives, the local FAIRification team will be able to make smarter choices that are aligned with the goals. Additionally, by understanding the FAIRification context and aims, the team can be more motivated to go through the implementation process.
- *Have access to FAIR experts.* FAIRification knowledge is complex and multi-faceted, which raises the need to establish the connections of standards and tools with the FAIRification workflow. For that purpose, a network of experts specialised in FAIRification of research data is needed. Access to such a wealth of expertise also aided the FAIR data stewards in the development of guidelines.
- *Attend active training about FAIR(ification).* While collecting the FAIRification challenges, we realised that there was a significant difference in the perception of FAIR between the different ERNs. Some were unfamiliar with the FAIR principles, while others had different interpretations of them. As a result of this knowledge gap, some ERNs may have faced similar challenges but articulated them differently. To reach a consensus on FAIR literacy as well as FAIR awareness, attending workshops and hackathons to share experiences and brainstorm ideas is of foremost importance.
- *Use the Common Data Elements (CDEs) and their semantic data model.* Collecting the CDEs can increase interoperability among ERNs, but, even if a pre-specified list of CDEs is provided, there are still many challenges regarding compliance and interpretation with that list. Thus, representing these CDEs through a semantic data model in a machine-readable fashion is needed to reduce ambiguity. Further, since the CDEs do not capture various domain-specific data elements, a new list of Domain-specific Common Data Elements (DCDEs) is being developed by the EJP RD to be applied to the RD registries.
- *Have vendors incorporate FAIRification in the data collection software.* ERN registries are dependent on various software to collect and manage their data. When a FAIRification workflow is embedded in the registry data collection process, the burden of making data FAIR is reduced.
- *Define and reuse community standards.* Standards and implementation choices should be defined and reused within the related research community to converge and harmonise by default.
- *Resolve legal issues internationally in a FAIR optimised way.* By legal issues, we refer to pseudonymisation, informed consent, data processing agreements and data sharing policies. Any disagreements between these can become the bottleneck that hinders many steps of FAIRification and drags out the entire process. In addition, tackling these issues is time-consuming and labour intensive but still necessary, which requires dedicated negotiations across countries.

### Future work

At this date, large efforts have been deployed to support ERNs with the CDEs implementation and FAIRification. In the second year of work, the FAIR data stewards collected and compared ERNs’ data dictionaries to identify common research, disease, or domain-specific data elements (DCDEs). The goal of DCDEs is to reach convergence and standardisation of what and how ERNs collect data elements other than CDEs, thereby increasing interoperability, facilitating collaborative research, and improving data discoverability. An additional advantage is that the newly identified commonly collected data elements will be semantically modelled by EJP RD experts in close collaboration with the domain experts who are choosing the DCDEs. Separating these processes and the modellers from the domain experts, as was the case for the CDEs, makes accurate modelling much harder. They are also expected to be added to ERDRI to encourage reuse by new RD registries.

The challenges presented in this study were collected as one of the FAIR data stewards’ initial tasks. In the future, we plan to further support FAIRification, by providing a Smart Guidance tool. This tool will combine the knowledge, results, and resources of the FAIR data stewards and EJP RD FAIR, and create an interactive questionnaire that generates a personalised FAIRification plan. A partial preview of the Smart Guidance content can be found in the visual representation called FAIRopoly [43].

The FAIR data stewards will continue to support the FAIRification of ERN registries. We will first reassess the implementation status of standards and tools used in the ERNs registries' FAIRification to learn the effect of our FAIR guidance and proposed solutions. We also plan to support the FAIRification of other EJP RD resources.

## Conclusion

We identified the main challenges faced by RD registries during FAIRification and proposed collaborative solutions to address them. ERNs desire to learn about EJP RD-recommended tools and standards for facilitating FAIRification, and have a high demand for assistance in implementing these tools and standards. This overview is a valuable resource for EU-wide FAIRification efforts in the RD field. For example, the most common challenge may be the most significant bottleneck, and therefore it might be prioritised in most FAIRification initiatives.

As FAIR data stewards, we supported the harmonisation of solutions for making RD data FAIR across countries, and continue to futnction sustainably, as motivated by the work described in this paper. We anticipate that our findings and lessons learned will increase FAIR awareness in the RD field and provide suggestions for other large FAIRification efforts. Specifically, we foresee that our unique team-based setup for supporting FAIRification will be adopted by other projects to recreate a similar hovering consultant team.

## Supplementary information

**Additional file 1.**. Template of implementation document.

**Additional file 2.**. Complete challenge-solution matrix.

## Data Availability

The data that support the fndings of this study are either included in the article (or in its supplementary files) or available from the corresponding author on reasonable request.

## Abbreviations

RD: Rare disease
CDEs: Common data elements
EDC: Electronic data capture
ERN: European reference network
FAIR: Findable, accessible, interoperable, and reusable
JRC: Joint research centre
WHODAS: WHO disability assessment schedule
ERICA: European rare disease research coordination and support action
PROs: Patient-reported outcomes
ERDRI: the European rare disease registry infrastructure
